# Anesthetic and airway management for inguinal hernia repair in a pediatric patient with fibrodysplasia ossificans progressiva: a case report

**DOI:** 10.1186/s13256-025-05756-4

**Published:** 2026-01-28

**Authors:** Saori Ozaki, Satoshi Kamiya, Noriko Takeno, Tomomi Ishii, Yukari Toyota, Hiroshi Yokomi, Takahiro Kato, Yasuo M. Tsutsumi

**Affiliations:** Department of Anesthesiology and Critical Care, Hiroshima University, 1-2-3 Kasumi, Minami-Ku, Hiroshima, 734-8551 Japan

**Keywords:** Fibrodysplasia ossificans progressiva, Pediatric anesthesia, Difficult airway, Fiberoptic intubation

## Abstract

**Background:**

Fibrodysplasia ossificans progressiva is a rare genetic disorder characterized by the progressive heterotopic ossification of soft connective tissues, ultimately leading to cumulative disability and ankylosis. Airway management during general anesthesia in patients with fibrodysplasia ossificans progressiva is particularly challenging because of anatomical abnormalities and the risk of triggering flare-ups due to minor trauma. However, reports on the anesthetic management of pediatric patients remain limited.

**Case presentation:**

We present the case of a 10-year-old Japanese girl with genetically confirmed fibrodysplasia ossificans progressiva who underwent an uneventful elective inguinal hernia repair under general anesthesia. She had hallux valgus at birth and was diagnosed with fibrodysplasia ossificans progressiva at the age of 2 years. Preoperative imaging revealed temporomandibular joint ankylosis with limited mouth opening (interincisal distance ~2 cm), cervical spine fusion, and thoracic heterotopic ossification. A high-flow nasal cannula was used for preoxygenation and apneic oxygenation. Mask ventilation was confirmed to be adequate after induction with remifentanil and propofol. Gentle mandibular elevation and nasally guided fiberoptic intubation were successfully performed while maintaining peripheral oxygen saturation levels above 99%. Anesthesia was maintained using remifentanil and sevoflurane. Perioperative corticosteroids and nonsteroidal anti-inflammatory drugs were administered to prevent flare-ups. The surgery lasted 82 minutes, and the patient was discharged uneventfully the following day without any signs of flare-up or airway complications.

**Conclusion:**

General anesthesia in pediatric patients with fibrodysplasia ossificans progressiva requires meticulous planning to address the dual challenges of difficult airway management and the prevention of flare-ups. This case demonstrates that nasal fiberoptic intubation under high-flow nasal cannula oxygenation, combined with pharmacological prophylaxis and atraumatic handling, can contribute to safe anesthetic care. This patient case contributes to the limited body of literature on pediatric fibrodysplasia ossificans progressiva anesthesia, underscoring the practical strategies for managing the airway and minimizing the risk of flare-ups.

## Background

Fibrodysplasia ossificans progressiva (FOP) is a rare genetic disorder characterized by progressive heterotopic ossification of soft connective tissues, leading to cumulative and irreversible disability. Its incidence is estimated to be approximately 1 in 2 million individuals. Classic FOP is caused by a recurrent mutation in Activin A receptor type I, also known as Activin receptor-like kinase 2, which encodes a bone morphogenetic protein type I receptor and is specific to the disorder [1].

Patients with FOP typically develop congenital hallux valgus. Within the first decade of life, they develop flare-ups and painful soft tissue swellings, which often progress to heterotopic ossification. Minor trauma, intramuscular injections, or viral infections can trigger these episodes. Ossification is irreversible and cumulative, leading to progressive joint immobility and, ultimately, total ankylosis. The initial manifestations often include temporomandibular joint ankylosis and cervical spine fusion, followed by reduced thoracic compliance, which predisposes patients to restrictive lung disease and secondary cardiovascular complications. Progressive ossification occurs throughout the body, and many patients become wheelchair-bound by their 30s, requiring lifelong assistance with activities of daily living. The estimated average life expectancy is 56 years, with respiratory failure being the most common cause of death. These anatomical and physiological factors make airway management and anesthetic care particularly challenging in patients with FOP. Preoperative planning must consider the limited range of motion and anatomical abnormalities resulting from existing ossified lesions. All procedures should be performed with minimal invasiveness to avoid provoking new ossifications or triggering flare-ups.

Owing to the extreme rarity of this condition, literature on anesthetic management in FOP remains scarce, especially in the pediatric population. Here, we report the anesthetic management of a pediatric patient with FOP and limited mouth opening who underwent inguinal hernia repair under general anesthesia.

## Case presentation

We present the case of a 10-year-old Japanese female patient (138 cm, 35 kg) with FOP. She had bilateral hallux valgus at birth and was diagnosed with FOP at 2 years of age. She developed a left inguinal hernia with a risk of bowel incarceration and was scheduled to undergo elective repair using the Potts technique [2]. There was no notable past medical history, family history, social background, or potential challenges other than FOP.

**Fig. 1:**
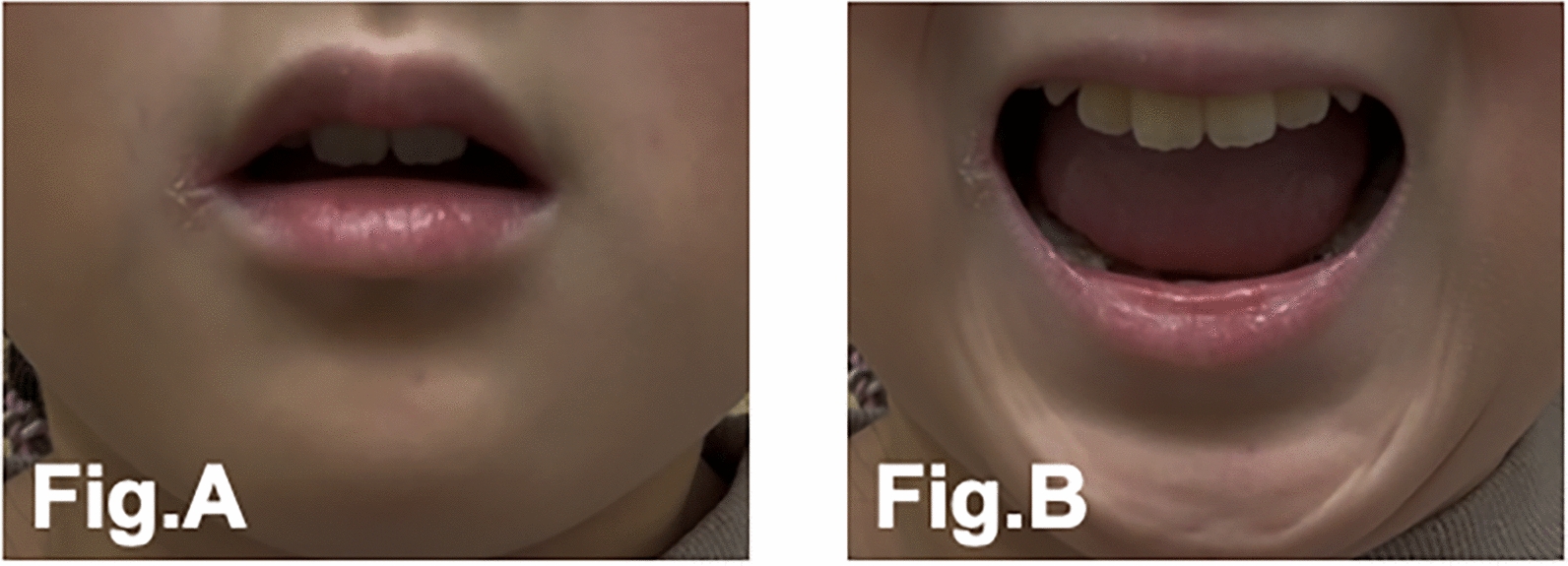
Limited mouth opening (left, **A**; right, **B**). **A** shows the patient’s maximal mouth opening without temporomandibular joint pain, with an interincisal distance of approximately 10 mm. **B** shows the maximum mouth opening within the patient’s functional range of motion with an interincisal distance of approximately 20 mm

**Fig. 2:**
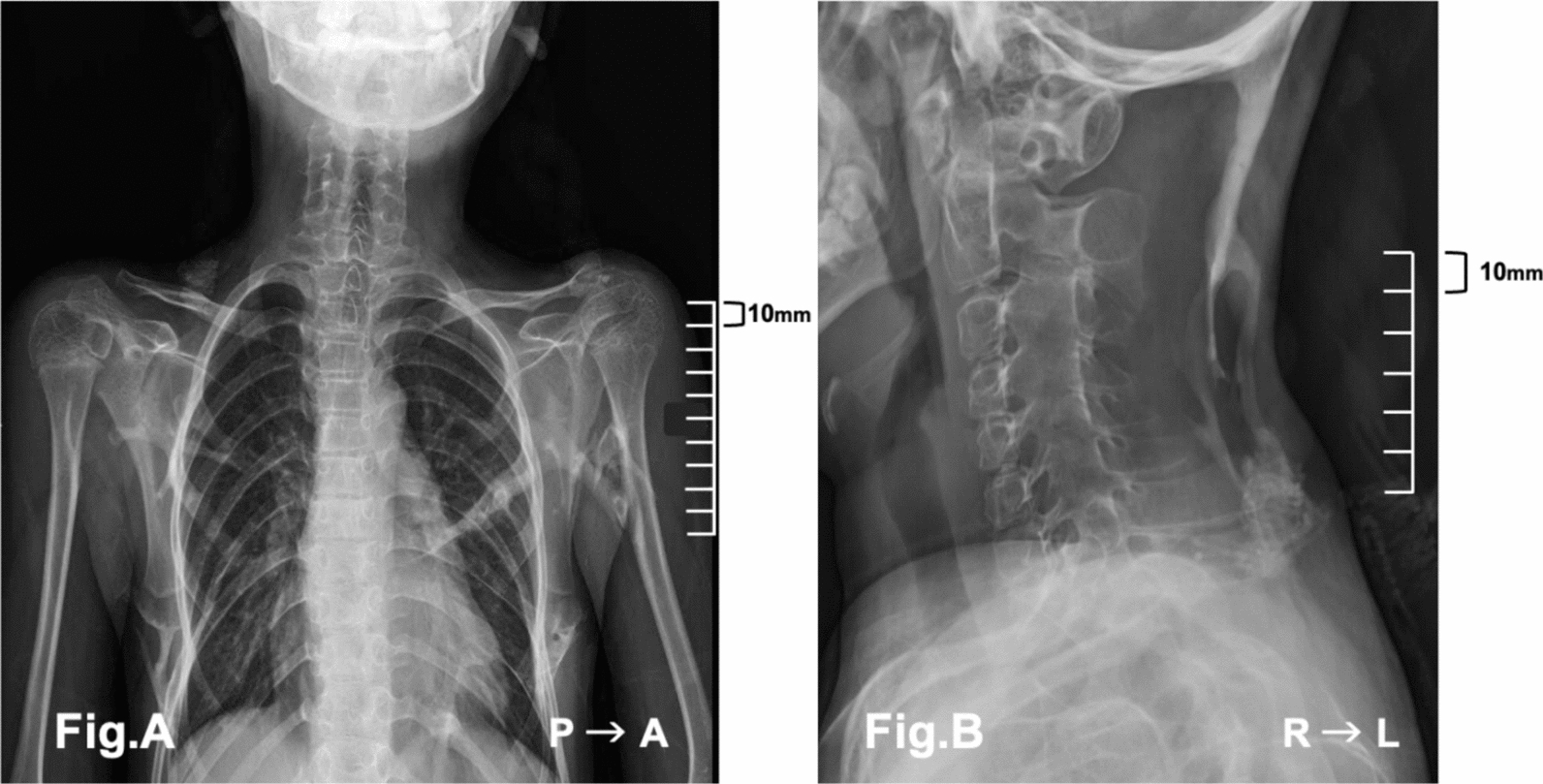
**A** Frontal chest radiograph. Preoperative imaging revealed cervical spine fusion and heterotopic ossification in the soft tissue adjacent to the inferior scapular border. However, no definitive thoracic cage deformity was evident at this stage. **B** Lateral cervical spine radiograph. Bony fusion was observed between the transverse and spinous processes of C2–C7. Subcutaneous heterotopic ossification was noted posterior to the C7 and T1 spinous processes. Additional ossification was observed between vertebral bodies, with irregularly shaped margins

Physical examination revealed marked ankylosis of the temporomandibular joints, with a maximal interincisal distance of approximately 2 cm and a Mallampati classification of grade IV (Fig. 1A, B). The neck was fixed in a neutral position for cervical spinal fusion. Despite limited mandibular mobility, partial jaw protrusion and lower-limb movements were preserved. Progressive heterotopic ossification was evident in the paravertebral soft tissues. Chest radiography revealed a heterotopic ossification involving the thoracic cage and shoulder girdle. Tracheal and spinal deviations were mild (Fig. 2A, B). Nutritional status was good, and oral intake remained unaffected. Laboratory findings were unremarkable except for mild leukocytosis. The patient clearly understood her illness and cooperated fully during the perioperative period.

**Fig. 3:**
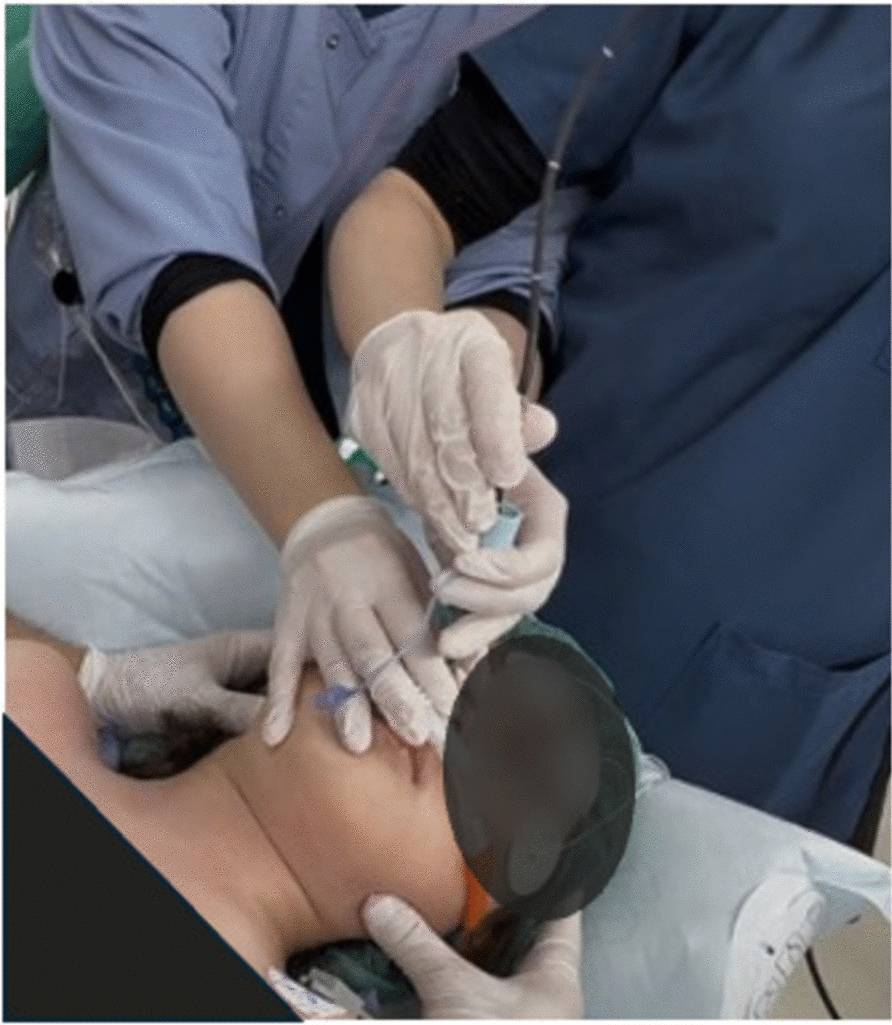
Manual in-line neck stabilization by assistants. The first assistant held the endotracheal tube, while the second assistant performed manual in-line neck stabilization to protect the cervical spine. Each assistant used both hands to prevent inadvertent external stimulation of the cervical spine

On the day of surgery, no premedication was administered. In the operating room, the patient was placed in a supine position with joint protection using cushions and a warm-air mattress. A 22G intravenous line was secured in the right hand. Preoxygenation was performed using a high-flow nasal cannula (HFNC; 30 L/minute, fraction of inspired oxygen [FiO₂] 1.0). Sedation was initiated with a continuous infusion of remifentanil at 0.1 µg/kg/minute and propofol at 1 mg/kg/hour. After confirming the loss of consciousness with preserved spontaneous respiration, manual in-line neck stabilization was performed (Fig. 3). The HFNC was temporarily removed to assess the feasibility of mask ventilation, which was confirmed to be adequate. Rocuronium (0.6 mg/kg) was administered to induce neuromuscular blockade. The doses of remifentanil and propofol were increased (0.3 µg/kg/minute and 2 mg/kg/hour), respectively, and mask ventilation was continued. Following confirmation of neuromuscular blockade, HFNC oxygenation was resumed via one nostril, whereas a nasotracheal fiberoptic bronchoscope was inserted through the other. Although elevation of the epiglottis was challenging, mandibular advancement enabled the visualization of the vocal cords. The endotracheal tube was advanced under fiberoptic guidance without desaturation (peripheral oxygen saturation maintained at 99–100%). Anesthesia was maintained with remifentanil (0.3 µg/kg/minute) and sevoflurane (1.2–2%). Analgesia was achieved with fentanyl (175 µg total) and intravenous acetaminophen (525 mg). Perioperative corticosteroids and cyclooxygenase inhibitors were administered to prevent flare-ups. The surgery lasted for 82 minutes. After spontaneous breathing was confirmed, the patient was extubated and returned to the general ward with stable vital signs. The patient was discharged on the following day. No flare-ups were observed in the jaw, neck, or oral cavity. Small induration at the surgical site was noted as the only complication. At the outpatient follow-up visit 2 months later, no new flare-ups were identified, and the localized induration at the wound site remained unchanged.

## Discussion

FOP is a rare disorder characterized by the progressive heterotopic ossification of soft tissues, including the airways and respiratory musculature. In addition to the limited mobility of the anatomical structures that are critical for airway management, even minor trauma can trigger flare-ups and accelerate disease progression. Comprehensive multidisciplinary planning is essential for the safe administration of general anesthesia in patients with FOP. Reports on general anesthesia for surgical procedures in pediatric patients are limited [3–5]. In particular, case reports that focus on anesthetic management, including drug selection, and images depicting airway management are rare. This patient case provides a valuable addition to the literature on the safe management of anesthesia. The difficulties associated with general anesthesia in patients with FOP can be primarily attributed to two major factors.

First, airway management is challenging. This patient exhibited cervical spine fusion and restricted mouth opening. Even among pediatric patients, the extent of disease progression and distribution of heterotopic ossification vary individually [6], necessitating tailored airway strategies. In this patient, the ability to perform manual mask ventilation was evaluated following the induction of unconsciousness with intravenous sedatives. This assessment was deemed essential because the feasibility of mask ventilation critically influences the selection of airway management strategies. Furthermore, preoxygenation and apneic oxygenation using HFNC effectively prevent hypoxemia [7]. HFNC reduces dead space and breathing, which is beneficial for preserving spontaneous ventilation [8]. Upper airway abnormalities combined with thoracic deformities may contribute to significant ventilatory challenges. Airway obstruction and even cardiac arrest during induction have been reported [4, 5], emphasizing the importance of adequate oxygenation and monitoring. Novel monitoring tools such as the oxygen reserve index and acoustic respiratory rate have been reported as useful [8]. In cases with restrictive ventilation impairment due to chest wall deformities, intraoperative ventilation assessment may be facilitated using electrical impedance tomography, which offers a noninvasive and dynamic evaluation of ventilation [9].

The second is flare-up prevention. Corticosteroids, cyclooxygenase-2 inhibitors, and nonsteroidal anti-inflammatory drugs (NSAIDs) have anti-inflammatory effects and may be useful for the prevention of flare-ups as well as for symptomatic treatment in the early stages of a flare-up. Patients with FOP may develop heterotopic ossification even after regional anesthesia or intramuscular injections. General anesthesia is commonly used for procedures requiring sedation in patients with FOP. Inhalational anesthesia with sevoflurane may cause unexpected vigorous body movements due to its characteristic excitation phase. Additionally, during bronchoscopic procedures under spontaneous respiration, the alveolar concentration of inhaled anesthetics can become unstable. Therefore, in this individual, we selected intravenous anesthesia using propofol and remifentanil. In the preoperative setting, both joint mobility and individualized positioning based on the symptoms of the patient should be considered to minimize pain and prevent flare-ups. Nasal fiberoptic intubation is recommended for airway management. Sedated nasal fiberoptic intubation is considered appropriate for pediatric patients or those who are uncooperative. The use of direct laryngoscopy can lead to overextension of the temporomandibular joint and potentially trigger flare-ups and should be avoided even in patients without trismus [3]. According to Kaplan *et al*., video laryngoscopes such as the GlideScope^®^ are advisable for patients with restricted mouth opening, as they facilitate intubation under limited oral access conditions [10]. Similarly, the use of supraglottic airway devices may pose a risk of flare-ups and should be approached with caution. In emergency settings, surgical airway management may be necessary. However, ossification at the incision site can lead to airway obstruction, and such procedures should be avoided whenever possible. If tracheostomy is inevitable and anterior neck ossification is present, surgical drills may be required [10], making preoperative imaging indispensable.

General anesthesia for patients with FOP requires careful airway planning, considering difficult airways and ventilatory compromise, as well as minimally invasive techniques to prevent flare-ups. This patient case demonstrates that a multifaceted approach can enable safe anesthetic management.

## Conclusion

Here, we describe the anesthetic management of a pediatric patient with FOP, emphasizing the importance of individualized multidisciplinary planning. Atraumatic airway strategies, including nasally guided fiberoptic intubation under high-flow nasal oxygenation combined with perioperative anti-inflammatory prophylaxis, can help minimize the risk of disease exacerbation and contribute to safe perioperative outcomes in patients with FOP. This patient case illustrates that meticulous patient-specific strategies are essential for achieving safe general anesthesia in this rare and high-risk population.

## Data Availability

The data described in this study are available upon request from the authors and are anonymized to protect patient identity.

## Abbreviations

FOP: Fibrodysplasia ossificans progressiva
IV: Intravenous
HFNC: High-flow nasal cannula
